# Nomogram based on homogeneous and heterogeneous associated factors for predicting bone metastases in patients with different histological types of lung cancer

**DOI:** 10.1186/s12885-019-5445-3

**Published:** 2019-03-15

**Authors:** Chao Zhang, Min Mao, Xu Guo, Ping Cui, Lianmin Zhang, Yao Xu, Lili Li, Xiuxin Han, Karl Peltzer, Shunbin Xiong, Vladimir P. Baklaushev, Xin Wang, Guowen Wang

**Affiliations:** 1 Department of Bone and Soft Tissue Tumors, Tianjin Medical University Cancer Institute and Hospital, National Clinical Research Center for Cancer, Key Laboratory of Cancer Prevention and Therapy, Tianjin’s Clinical Research Center for Cancer, Tiyuan Bei Road, Hexi District, Tianjin, China; 2 Department of Epidemiology and Biostatistics, First Affiliated Hospital, Army Medical University, 30 Gaotanyan Street Shapingba District, Chongqing, China; 3 Department of Lung Cancer, Tianjin Medical University Cancer Institute and Hospital, National Clinical Research Center for Cancer, Key Laboratory of Cancer Prevention and Therapy, Tianjin’s Clinical Research Center for Cancer, Tianjin, China; 4 Department of Pathology and Southwest Cancer Center, Southwest Hospital, Third Military Medical University, Chongqing, China; 5 Department of Research and Innovation, University of Limpopo, Turfloop, South Africa; 6 Department of Genetics, M.D. Anderson Cancer Center, The University of Texas, Houston, TX USA; 7 Department of Orthopedics, Cangzhou Central Hospital, Cangzhou, Hebei China; 8 Department of Epidemiology and Biostatistics, Tianjin Medical University Cancer Institute and Hospital, Tianjin, China; 9 Federal Research and Clinical Center of Specialized Medical Care and Medical Technologies, Federal Biomedical Agency of the Russian Federation, Moscow, Russian Federation

**Keywords:** Lung cancer, Metastasis, SEER, Screening

## Abstract

**Background:**

The purpose of the present study was to characterize the prevalence, associated factors, and to construct a nomogram for predicting bone metastasis (BM) with different histological types of lung cancer.

**Patients and methods:**

This study was a descriptive study that basing on the invasive lung cancer patients diagnosed between 2010 and 2014 in Surveillance, Epidemiology, and End Results program. A total of 125,652 adult patients were retrieved. Logistic regression analysis was conducted to investigate homogeneous and heterogeneous factors for BM occurrence. Nomogram was constructed to predict the risk for developing BM and the performance was evaluated by the receiver operating characteristics curve (ROC) and the calibration curve. The overall survival of the patients with BM was analyzed using the Kaplan–Meier method and the survival differences were tested by the log-rank test.

**Results:**

A total of 25,645 (20.9%) were reported to have BM, and the prevalence in adenocarcinoma, squamous cell carcinoma, small cell lung cancer (SCLC), large cell lung cancer (LCLC), and non-small cell lung cancer/not otherwise specified lung cancer (NSCLC/NOS) were 24.4, 12.5, 24.7, 19.5 and 19.4%, respectively, with significant difference (*P* < 0.001). Male gender, more metastatic sites and lymphatic metastasis were positively associated with BM in all lung cancer subtypes. Larger tumor size was positively associated with BM in all the lung cancer subtypes except for NSCLC/NOS. Poorly differentiated histology was positively associated with adenocarcinoma, squamous cell carcinoma and NSCLC/NOS. The calibration curve and ROC curve exhibited good performance for predicting BM. The median survival of the bone metastatic lung cancer patients was 4.00 (95%CI: 3.89–4.11) months. With the increased number of the other metastatic sites (brain, lung and liver metastasis), the survival significantly decreased (*p* < 0.001).

**Conclusion:**

Different lung cancer histological subtypes exhibited distinct prevalence and homogeneity and heterogeneity associated factors for BM. The nomogram has good calibration and discrimination for predicting BM of lung cancer.

**Electronic supplementary material:**

The online version of this article (10.1186/s12885-019-5445-3) contains supplementary material, which is available to authorized users.

## Background

Lung cancer is the most common carcinoma and the leading cause of cancer death globally [1]. Patients with metastatic disease continue to exhibit a poor prognosis [2]. Bone metastasis (BM) was reported to occur in 15–40% of lung cancer patients. The median survival time of patients with BM was reported to be less than one year [3–5].

Early diagnosis and intervention in patients with BM could significantly influence the survival rate of patients [6, 7]. However, the Lung Cancer National Comprehensive Cancer Network (NCCN) screening guidelines do not recommend performing routine assessment or continued reassessment for BM by skeletal imaging in asymptomatic patients [8]. Previous studies reported a series of associated factors for BM, which provided the basis for predicting the BM risk [9, 10]. To promptly perform metastatic screening, a predictive nomogram based on the clinicopathologic features of lung cancer is warranted.

With the development of tumor homogeneity/heterogeneity theory, different histological types of lung cancer are now recognized to exhibit distinct prevalence of BM [4, 11]. We also believe that different histological types of lung cancer are associated with different factors for BM occurrence. Further study identifying the homogeneous and heterogeneous BM associated factors could help physicians to specifically identify the BM risk for different types of lung cancer and tailor targeted preventive treatment strategies.

The purpose of the present study was to characterize the prevalence and associated factors for BM in patients with different histological types of lung cancer using the Surveillance, Epidemiology, and End Results (SEER) database. Meanwhile, the clinical factor based nomogram was built to predict the BM risk and potentially guide the BM screening.

## Methods

### Ethnics statement

Cancer is a reportable disease in every state of the United States. The data in the SEER database does not require informed patient consent. The present study was complied with the 1964 Helsinki Declaration and its later amendments or comparable ethical standards. This study used previously collected deidentified data, which was deemed exempt from review by the Ethics Board of the Tianjin Medical University Cancer Institute and Hospital.

### Data source

It was a population based descriptive study and the data were abstracted from the SEER 18 registries research database, comprising approximately 30% of the total US population. As the data for metastatic sites of bone, liver, lung and brain were not collected until 2010, lung cancer patients who were diagnosed between 2010 and 2014 were included in the present study. SEER*Stat Software version 8.3.4 (https://seer.cancer.gov/seerstat/) (Information Management Service, Inc. Calverton, MD, USA) was used to generate the case listing.

### Cohort selection

**Fig. 1:**
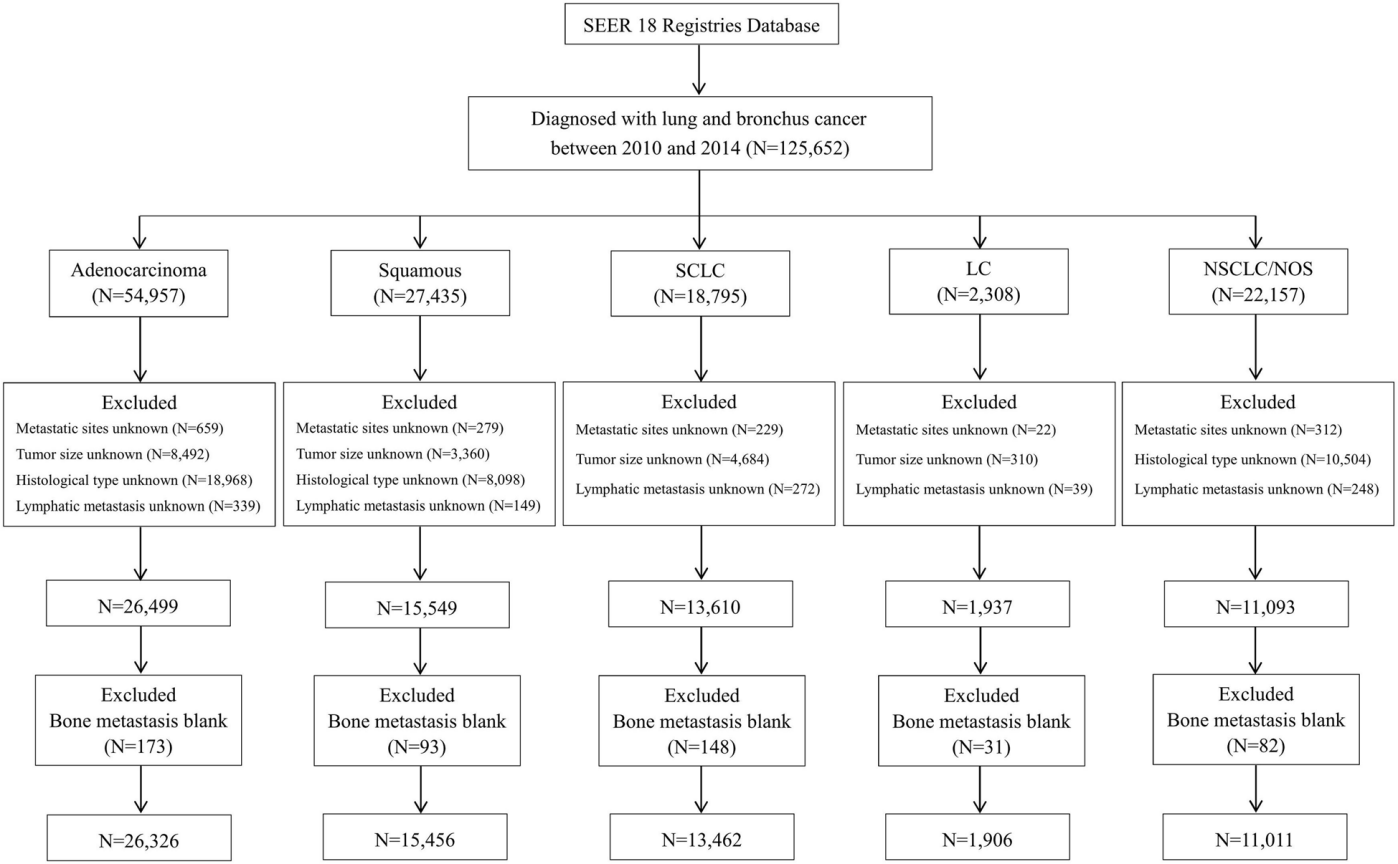
Flow-chart for different histological types of lung cancer patients selection. SCLC = small cell lung cancer; LC = large cell; NSCLC/ NOS = non-small cell lung cancer/not otherwise specified

The inclusion criteria were as follows: (1) aged 18–79 years; (2) diagnosed as the first and only malignant cancer; (3) only one primary site; (4) American Joint Committee on Cancer (7th edition) stage I-III; and (5) diagnosis not obtained from a death certificate or an autopsy. Patients diagnosed before 2010 were excluded because the SEER did not record BM data until 2010. The lung cancer was histologically classified as adenocarcinoma, squamous cell carcinoma, small cell lung cancer (SCLC), large cell lung cancer (LCLC) and non-small cell lung cancer or not otherwise specified lung cancer (NSCLC/NOS) based on the International Classification of Diseases for Oncology, 3rd Edition codes. The flow-chart for the population selection was shown in Fig. 1.

### Statistical analysis

Quantitative data were described as mean ± standard deviation (SD) and the difference between groups were analysed by student’s t-test. Categorical data was presented as number and the percentage (N, %). Pearson chi-square test was used to evaluate the difference between categorical variables. The univariable and multivariable logistic regression model were conducted to determine the associated factors of BM by different histological types of lung cancer. Factors with a *P*-value less than 0.05 were incorporated into the multivariable regression model. The final model selection was performed by a backward step-down selection process using the Akaike information criterion.

A nomogram was also formulated based on the results of multivariable logistic analysis using the rms package in R version 3.4.1 (R Foundation for Statistical Computing, Vienna, Austria; www.r-project.org). The performance of the nomogram was evaluated by the receiver operating characteristics (ROC).

To evaluate the calibration of the nomogram, a regression smoothing method was used to produce the calibration plots by bootstrapping with 1000 resamples, where the relationship between the observed and predicted probabilities of BM was described graphically.

Randomly splitting and the temporal splitting method were used to evaluate the stability of the nomogram. An ROC curve was constructed to evaluate the performance of the construction and validation model, and the difference in the aura under the curve (AUC) was tested by DeLong’s test. Statistically significant levels were two-tailed and set at *P* < .05. Statistical analyses were performed using the statistical Package for the Social Sciences (SPSS) version 23.0 software package for Windows (SPSS, Inc.).

The overall survival of the lung cancer patients with bone metastasis were analyzed using the Kaplan–Meier method and the survival difference between different metastatic sites, treatment regimens of surgery, radiotherapy and chemotherapy were tested by the log-rank test.

## Results

### Patient characteristics

The study consisted of 125,652 patients, including 54,957 (43.7%) cases of adenocarcinoma, 27,435 (21.8%) of squamous cell carcinoma, 18,795 (15.0%) of SCLC, 2308 (1.8%) of LCLC and 22,157 (17.6%) of NSCLC/NOS lung cancer patients. Among them, 67,216 (53.5%) were male, and 58,436 (46.5%) were female, mean age was 64.84 ± 9.17 years (Table 1).

**Table 1:** Distribution of demographic and clinical information on different histological types of lung cancer

Factors	Total population (*N* = 125,652; 100.0%)		Adenocarcinoma (*N* = 54,957; 43.7%)		Squamous (*N* = 27,435; 21.8%)		SCLC (*N* = 18,795; 15.0%)		LC (*N* = 2308; 1.8%)		NSCLC/NOS (*N* = 22,157; 17.6%)	
	*N*	*%*	*N*	*%*	*N*	*%*	*N*	*%*	*N*	*%*	*N*	*%*
Bone metastasis												
No	97,246	77.4	40,639	73.9	23,571	85.9	13,780	73.3	1812	78.5	17,444	78.7
Yes	25,645	20.4	13,110	23.9	3377	12.3	4521	24.1	440	19.1	4197	18.9
Unknown/Blank	2761	2.2	1208	2.2	487	1.8	494	2.6	56	2.4	516	2.4
Age (years)												
18–45	3237	2.6	1647	3.0	317	1.2	287	1.5	67	2.9	919	4.1
46–65	57,701	45.9	26,423	48.1	10,789	39.3	9184	48.9	1172	50.8	10,133	45.7
> 65	64,714	51.5	26,887	48.9	16,329	59.5	9324	49.6	1069	46.3	11,105	50.1
Sex												
Female	58,436	46.5	27,645	50.3	9833	35.8	9337	49.7	968	41.9	10,653	48.1
Male	67,216	53.5	27,312	49.7	17,602	64.2	9458	50.3	1340	58.1	11,504	51.9
Race												
White	99,978	79.6	41,865	76.2	22,446	81.8	16,235	86.4	1862	80.7	17,570	79.3
Black	16,274	13.0	7576	13.8	3538	12.9	1792	9.5	342	14.8	3026	13.7
Asian or Pacific Islander	8393	6.7	5090	9.3	1213	4.4	618	3.3	88	3.8	1384	6.2
Indian/Alaska Native	669	0.5	248	0.5	178	0.6	124	0.7	10	0.4	109	0.5
Unknown/Blank	338	0.3	178	0.3	60	0.2	26	0.1	6	0.3	68	0.3
Marital status												
Unmarried	20,801	16.6	9125	16.6	4423	16.1	2977	15.8	418	18.1	3858	17.4
Married	99,001	78.8	43,246	78.7	21,727	79.2	15,027	80.0	1815	78.6	17,186	77.6
Unknown/Blank	5950	4.6	2586	4.7	1285	4.7	791	4.2	75	3.2	1113	5.0
Household income												
< 50,000$	42,788	34.0	16,295	29.7	10,897	39.7	7630	40.6	916	39.7	7050	31.8
50,000–80,000$	73,494	58.5	33,810	61.5	14,878	54.2	10,097	53.7	1256	54.4	13,453	60.7
> 80,000$	9362	7.5	4848	8.8	1657	6.0	1068	5.7	136	5.9	1653	7.5
Unknown/Blank	8	0.0	4	0.0	3	0.0	0	0.0	0	0.0	1	0.0
Insurance status												
Uninsured	5090	4.0	2243	4.1	946	3.4	832	4.4	113	4.9	956	4.3
Insured	118,612	94.4	51,877	94.4	26,105	95.2	17,679	94.1	2161	93.6	20,790	93.8
Unknown/Blank	1950	1.6	837	1.5	384	1.4	284	1.5	34	1.5	411	1.9
Metastatic sites												
0 site	81,989	65.3	35,126	63.9	21,447	78.2	9376	49.9	1433	62.1	14,607	66.0
1 site	31,981	25.4	14,275	26.0	4669	17.0	6944	36.9	642	27.8	5451	24.6
2 sites	8845	7.0	4234	7.7	902	3.3	1982	10.5	190	8.2	1537	6.9
3 sites	1336	1.1	663	1.2	138	0.5	264	1.4	21	0.9	250	1.1
Unknown/Blank	1501	1.2	659	1.2	279	1.0	229	1.2	22	1.0	312	1.4
Tumor size												
< 2 cm	16,948	13.5	8691	15.8	2557	9.3	1613	8.6	289	12.5	3798	17.1
2–5 cm	52,309	41.6	25,207	45.9	11,149	40.6	5982	31.8	914	39.6	9057	40.9
5–10 cm	31,367	25.0	11,076	20.2	9191	33.5	5349	28.5	649	28.1	5102	23.0
> 10 cm	4173	3.3	1166	2.1	1065	3.9	1047	5.6	135	5.8	760	3.5
Unknown/Blank	20,855	16.6	8817	16.0	3473	12.7	4804	25.6	321	13.9	3440	15.5
Histological type												
Well differentiated	6855	5.5	3970	7.2	530	1.9	27	0.1	3	0.1	2325	10.5
Moderate differentiated	20,707	16.5	11,145	20.3	7428	27.1	45	0.2	22	1.0	2067	9.3
Poor differentiated	32,932	26.2	14,570	26.5	9470	34.5	1688	9.0	780	33.8	6424	29.0
Undifferentiated	4332	3.4	300	0.5	167	0.6	2748	14.6	493	21.4	624	2.8
Unknown/Blank	60,826	48.4	24,972	45.4	9840	35.9	14,287	76.0	1010	43.8	10,717	48.4
Lymphatic metastasis												
N0	43,173	34.4	20,357	37.0	10,536	38.4	2521	13.4	784	34.0	8975	40.5
N1	10,881	8.7	4659	8.5	2839	10.3	1319	7.0	210	9.1	1854	8.4
N2	48,818	38.9	19,706	25.9	10,220	37.3	10,315	54.9	898	38.9	7679	34.7
N3	18,747	14.9	8311	15.1	3255	11.9	4029	21.4	347	15.0	2805	12.7
Unknown/Blank	4033	3.1	1924	3.5	585	2.1	611	3.3	69	3.0	844	3.8

Abbreviations: *SCLC* small cell lung cancer, *LC* large cell, *NSCLC/ NOS* non-small cell lung cancer/not otherwise specified

### Prevalence of BM

After excluding the patients with unknown BM information, 25,645 (20.9%) were reported to exhibit BM. When stratified by histological subtype, the prevalence of BM in adenocarcinoma, squamous cell carcinoma, SCLC, LCLC, and NSCLC/NOS were 24.4, 12.5, 24.7, 19.5 and 19.4%, respectively. The prevalence rates of BM in adenocarcinoma and SCLC were higher than those in the other lung cancer histological types (*P* < 0.001), and the prevalence of BM in squamous cell lung cancer was lowest among all types (*P* < 0.001) (Additional file 1: Table S1).

### Univariable logistic regression analysis

Advanced age [odds ratio (OR) = 0.81; 95% confidence interval (CI): 0.79–0.83; *P* < 0.001] and insured status (OR = 0.79; 95% CI: 0.74–0.85; *P* < 0.001) were negatively associated with BM. However, male gender (OR = 1.27; 95% CI: 1.23–1.31; *P* < 0.001), race (OR = 1.06; 95% CI: 1.03–1.08; *P* < 0.001), married status (OR = 0.95; 95% CI: 0.92–0.99; *P* = 0.005), household income (OR = 1.07; 95% CI: 1.05–1.10; *P* < 0.001), more metastatic sites (OR = 2.74; 95% CI: 2.69–2.80; *P* < 0.001), larger tumor size (OR = 1.29; 95% CI: 1.27–1.32; *P* < 0.001), poor histological differentiation (OR = 1.70; 95% CI: 1.65–1.75; *P* < 0.001), and more lymphatic metastasis (OR = 1.61; 95% CI: 1.59–1.63; *P* < 0.001) were positively associated with BM.

Additionally, diverse histological subtypes of the lung cancer exhibit a differential risk for developing BM. Compared with adenocarcinoma, squamous cell carcinoma (OR = 0.44; 95% CI: 0.43–0.46), LCLC (OR = 0.75; 95% CI: 0.68–0.84) and NSCLC/NOS (OR = 0.75; 95% CI: 0.72–0.78) were negatively associated with BM, whereas SCLC exhibited no difference in the risk of BM relative to adenocarcinoma (OR = 1.02; 95% CI: 0.98–1.06). Moreover, subgroup analysis suggested that the associated factors for bone metastases were not consistent across all the histological subtypes of lung cancer (Additional file 2: Table S2).

### Multivariable logistic regression analysis

When conducting multivariable logistic regression analysis, the patients with unknown or missing information concerning sex, metastatic site, tumor size, histological type and lymphatic metastasis were excluded. Finally, 68,161 patients with lung cancer were included (Fig. 1). For all the lung cancer patients, multivariable logistic regression suggested male gender, histological differentiation, more lymphatic metastasis, and lung cancer subtype were independent factors associated with BM (Additional file 3: Table S3). When stratified by the histological subtypes of lung cancer, results revealed that male gender, more metastatic sites, larger tumor size, poor histological differentiation and more lymphatic metastasis were all positively associated with BM in adenocarcinoma. The associated factors and OR with 95% CI for squamous cell carcinoma, SCLC, LCLC and NSCLC/NOS lung cancer are presented in Table 2.

**Table 2:** Multivariable logistic regression for analyzing the bone metastases associated factors in different histological types of lung cancer

Variable	Patients, *N*.		Entire cohort									
	No Bone metastasis *N* (%)	Bone metastasis *N* (%)	Adenocarcinoma		Squamous		SCLC		LC		NSCLC/NOS	
			OR (95% CI)	*P*-value	OR (95% CI)	*P*-value	OR (95% CI)	*P*-value	OR (95% CI)	*P*-value	OR (95% CI)	*P*-value
Sex												
Female	46,496 (47.8)	10,752 (41.9)	ref	1.0	ref	1.0	ref	1.0	ref	1.0	ref	1.0
Male	50,750 (52.2)	14,893 (58.1)	1.24 (1.15–1.34)	< 0.001	1.32 (1.15–1.51)	< 0.001	1.33 (1.23–1.45)	< 0.001	1.32 (1.01–1.72)	0.042	1.37 (1.21–1.55)	< 0.001
Metastatic sites												
0 site	71,363 (73.3)	10,192 (40.0)	ref	1.0	ref	1.0	ref	1.0	ref	1.0	ref	1.0
1 site	21,085 (21.7)	10,071 (39.6)	3.39 (3.11–3.70)	< 0.001	3.98 (3.47–4.57)	< 0.001	2.95 (2.69–3.23)	< 0.001	2.87 (2.16–3.81)	< 0.001	3.34 (1.91–3.82)	< 0.00`
2 sites	4339 (4.5)	4331 (17.0)	6.47 (5.68–7.36)	< 0.001	8.22 (6.40–10.54)	< 0.001	5.55 (4.89–6.30)	< 0.001	6.23 (4.22–9.21)	< 0.001	5.29 (4.34–6.45)	< 0.001
3 sites	439 (0.5)	868 (3.4)	12.08 (8.65–16.87)	< 0.001	13.64 (7.73–24.08)	< 0.001	7.57 (5.57–10.29)	< 0.001	5.97 (2.02–17.62)	0.001	10.38 (6.73–16.01)	< 0.001
Tumor size												
< 2 cm	14,785 (17.7)	1996 (10.0)	ref	1.0	ref	1.0	ref	1.0	ref	1.0	ref	1.0
2–5 cm	41,463 (49.7)	10,122 (51.0)	1.83 (1.60–2.11)	< 0.001	1.66 (1.23–2.25)	0.001	1.42 (1.22–1.65)	< 0.001	1.28 (0.82–2.00)	0.28	NS	NS
5–10 cm	23,894 (28.7)	6859 (34.6)	1.99 (1.72–2.31)	< 0.001	1.95 (1.44–2.64)	< 0.001	1.38 (1.19–1.61)	< 0.001	1.52 (0.97–2.40)	0.07	NS	NS
> 10 cm	3210 (3.9)	875 (4.4)	1.55 (1.21–2.00)	< 0.001	1.86 (1.26–2.73)	< 0.001	1.35 (1.10–1.66)	0.004	1.61 (0.87–2.97)	0.13	NS	NS
Histological type												
Well differentiated	6441 (11.8)	372 (4.1)	ref	1.0	ref	1.0	ref	1.0	ref	1.0	ref	1.0
Moderate differentiated	18,285 (33.4)	2149 (23.6)	1.49 (1.27–1.75)	< 0.001	0.81 (0.53–1.22)	0.31	NS	NS	NS	NS	2.31 (1.61–3.32)	< 0.001
Poor differentiated	26,632 (48.7)	5681 (62.4)	1.52 (1.30–1.78)	< 0.001	1.26 (0.84–1.88)	0.27	NS	NS	NS	NS	4.65 (3.36–6.42)	< 0.001
Undifferentiated	3315 (6.1)	909 (10.0)	1.80 (1.24–2.62)	< 0.001	2.05 (1.07–3.92)	0.03	NS	NS	NS	NS	4.56 (3.10–6.70)	< 0.001
Lymphatic metastasis												
N0	38,449 (40.4)	4325 (17.8)	ref	1.0	ref	1.0	ref	1.0	ref	1.0	ref	1.0
N1	8708 (9.2)	1985 (8.2)	1.77 (1.53–2.05)	< 0.001	1.82 (1.45–2.29)	< 0.001	1.30 (1.06–1.61)	0.013	2.29 (1.41–3.70)	0.001	1.99 (1.59–2.50)	< 0.001
N2	35,358 (37.2)	12,297 (50.6)	3.39 (3.07–3.75)	< 0.001	3.07 (2.61–3.61)	< 0.001	1.85 (1.60–2.13)	< 0.001	2.89 (2.05–4.08)	< 0.001	2.39 (2.04–2.80)	< 0.001
N3	12,635 (13.3)	5682 (23.4)	3.92 (3.46–4.45)	< 0.001	3.23 (2.62–3.99)	< 0.001	2.56 (2.20–2.99)	< 0.001	2.94 (1.94–4.45)	< 0.001	2.96 (2.43–3.61)	< 0.001

*SCLC* small cell lung cancer, *LC* large cell, *NOS* not otherwise specified, *NSCLC* non-small cell lung cancer, *NS* not significant

**Fig. 2:**
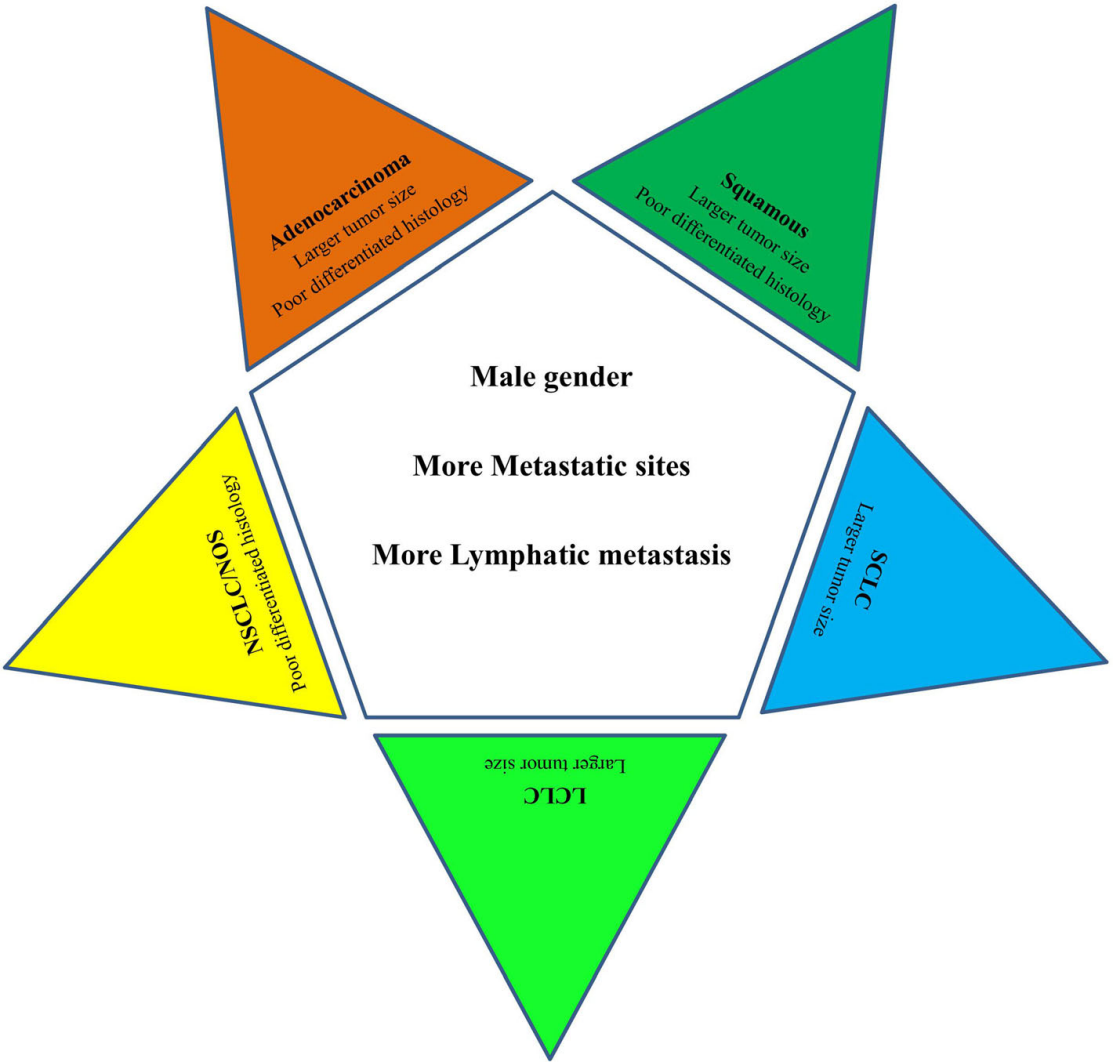
Homogeneous and heterogenous associated factors of bone metastasis in different histological subtypes of lung cancer. Factors of male gender, more metastatic sites and more lymphatic metastasis in the right pentagon were the homogeneous associated factors for bone metastasis for all the lung cancer subtypes. The factors listed in the angle exhibited the specific factors that associated with each histological lung cancer subtype

Different lung cancer histological subtypes exhibited homogeneity and heterogeneity for the factors associated with bone metastases. Male gender, more metastatic sites and more lymphatic metastasis were positively associated with bone metastases among all lung cancer histological subtypes. However, larger tumor size was not associated with BM in NSCLC/NOS. Poorly differentiated histology was positively associated with adenocarcinoma, squamous cell carcinoma and NSCLC/NOS lung cancer but not with SCLC or LCLC (Fig. 2).

### Performance of the nomogram for predicting BM

**Fig. 3:**
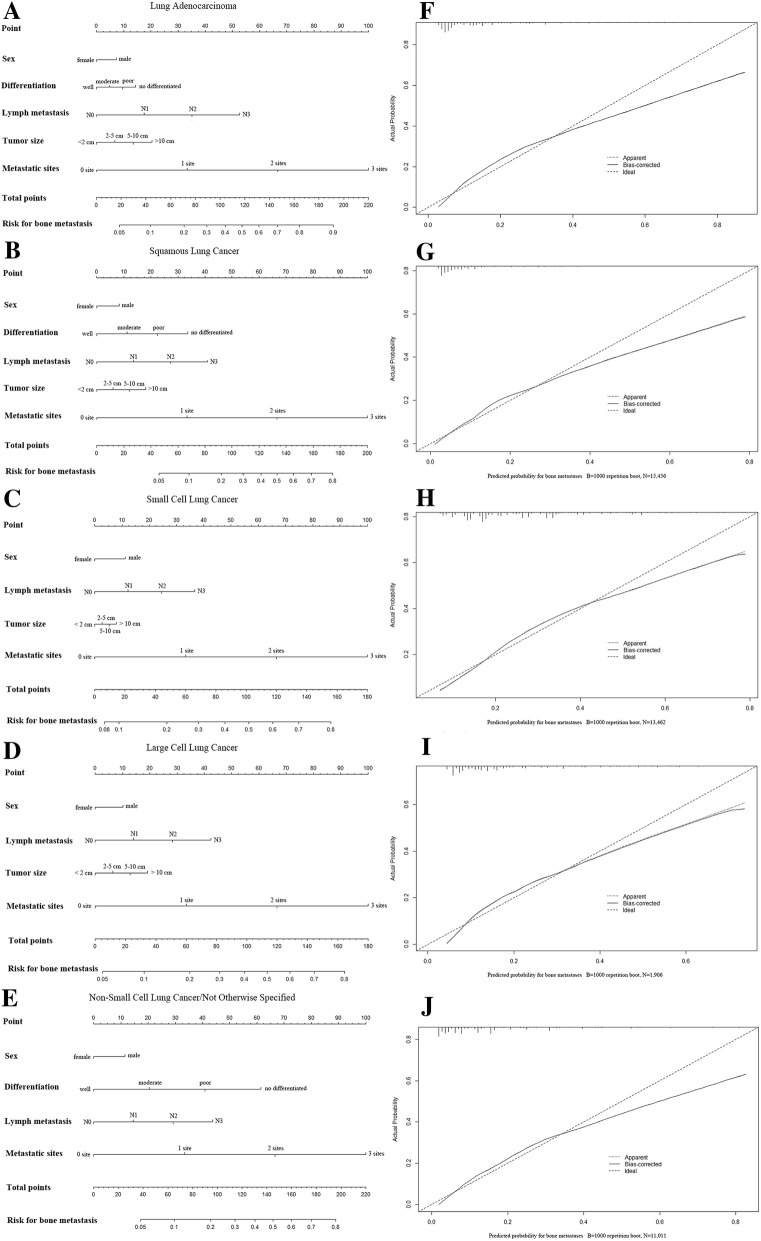
The predicting nomogram for bone metastasis in different histological subtypes of lung cancer and the curves for evaluating the calibration of each nomogram. **a-e**: nomogram for predicting the risk for developing bone metastasis of the adenocarcinoma, squamous cell carcinoma, small cell lung cancer, large cell lung cancer, and non-small cell lung cancer/not otherwise specified lung cancer, respectively. **f-j**: calibration curve for estimating the predictive accuracy for bone metastasis of the nomogram in adenocarcinoma, squamous cell carcinoma, small cell lung cancer, large cell lung cancer, and non-small cell lung cancer/not otherwise specified lung cancer, respectively

The prediction nomogram that integrated all significant factors for BM in different lung cancer histologic types is presented in Fig. 3. The calibration curve revealed good agreement between the predicted and observed probabilities for BM in different histological types of lung cancer. Moreover, the ROC curve of the nomogram exhibited good discrimination for predicting BM, and the AUC of the nomogram in adenocarcinoma, squamous cell carcinoma, SCLC, LCLC and NSCLC/NOS lung cancer were 80.3% (95% CI: 79.6–80.9%), 78.1% (95% CI: 76.8–79.4%), 70.8% (95% CI: 69.8–71.8%), 75.1% (95% CI: 72.3–77.8%) and 80.2% (95% CI: 79.2–81.3%), respectively.

### Validation of the nomogram

**Fig. 4:**
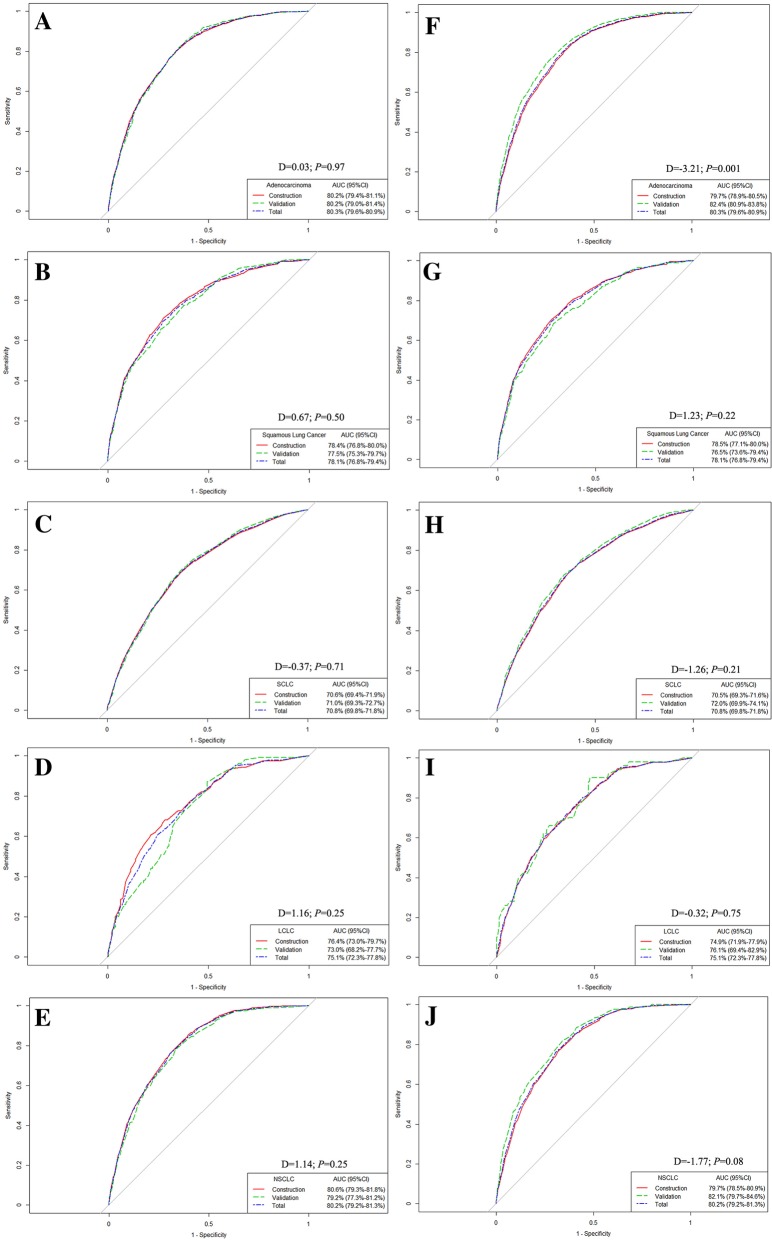
Internal validation of the stability of the predicting nomogram for different histological subtypes of lung cancer. **a-e**: randomly splitting method for evaluating the nomogram stability in adenocarcinoma, squamous cell carcinoma, small cell lung cancer, large cell lung cancer, and non-small cell lung cancer/not otherwise specified lung cancer, respectively. **f-j**: temporal splitting method for evaluating the nomogram stability in adenocarcinoma, squamous cell carcinoma, small cell lung cancer, large cell lung cancer, and non-small cell lung cancer/not otherwise specified lung cancer, respectively

For adenocarcinoma of lung cancer, the random splitting method revealed that the AUC values for the construction and validation model were 80.2% (95% CI: 79.4–81.1%) and 80.2% (95% CI: 79.0–81.4%), respectively, with no significant difference (D = 0.03; *P* = 0.97) (Fig. 4a). The nomogram for predicting BM was also stable in squamous cell carcinoma (D = 0.67; *P* = 0.50), SCLC (D = -0.37; *P* = 0.71), LCLC (D = 1.16; *P* = 0.25) and NSCLC/NOS (D = 1.14; *P* = 0.25) (Fig. 4b-e).

Temporal splitting analysis suggested that the AUC in the adenocarcinoma model to predict BM was 79.7% (95% CI: 78.9–80.5%), which was significantly lower than 280.9–83.8%) for patients diagnosed in 2014 (D = -3.21; *P* = 0.001) (Fig. 4f). However, there was no difference between the constructed and validation model for predicting BM in squamous cell carcinoma (D = 1.23; *P* = 0.22), SCLC (D = -1.26; *P* = 0.21), LCLC (D = -0.32; *P* = 0.75) or NSCLC/NOS (D = -1.77; *P* = 0.08), suggesting the stability of the predictive nomogram (Fig. 4g-j).

### Survival analysis for patients with BM

**Fig. 5:**
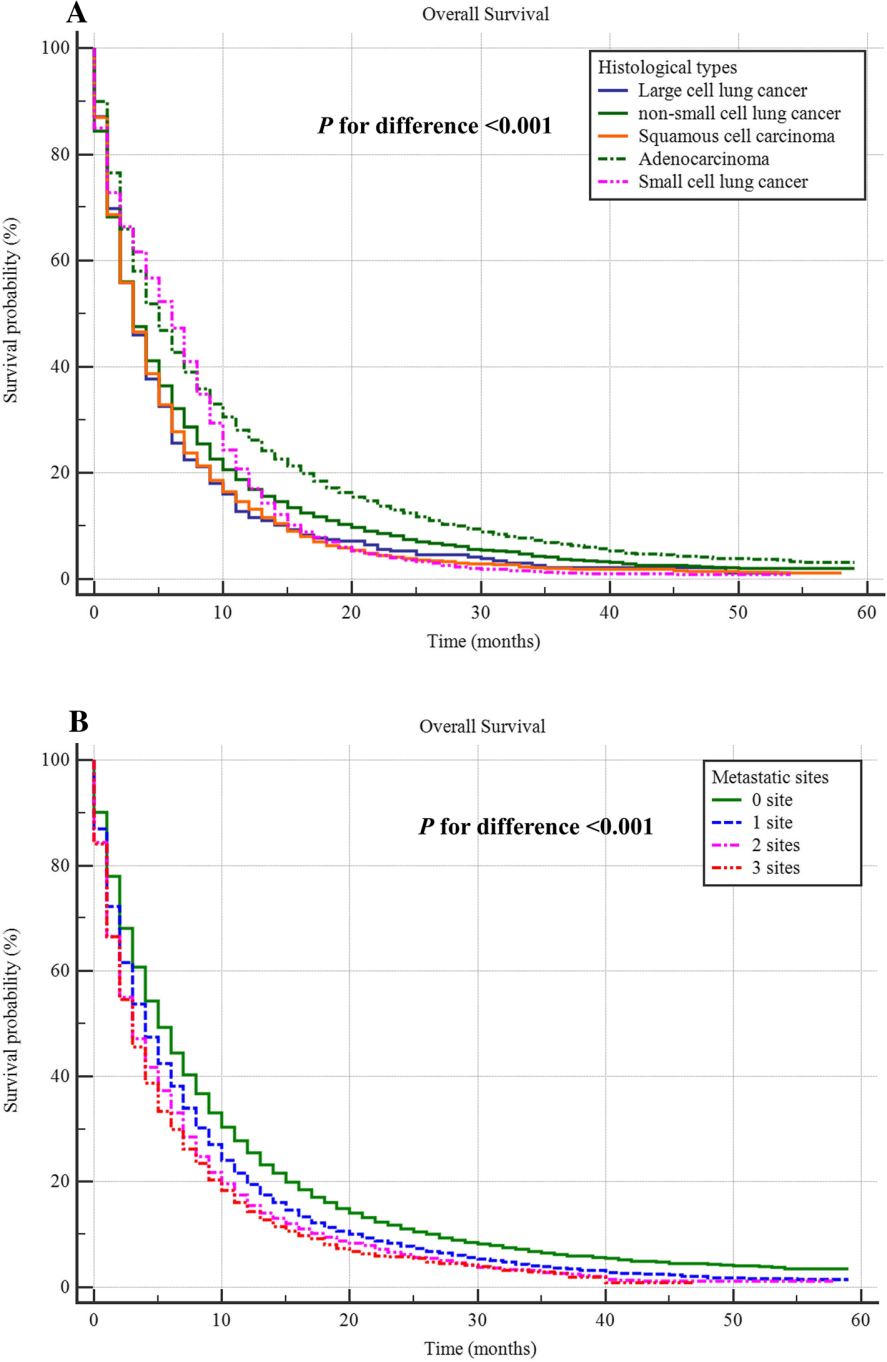
Survival curve for the difference among bone metastatic lung cancer patients with different histological types (**a**) and metastatic sites (**b**)

The median survival of the bone metastatic lung cancer patients was 4.00 (95%CI: 3.89–4.11) months. When stratified by different histological types, the median survival time for adenocarcinoma, squamous cell carcinoma, small cell lung cancer (SCLC), large cell lung cancer (LCLC), and non-small cell lung cancer/not otherwise specified lung cancer (NSCLC/NOS) were 5.00 (95%CI: 4.83–5.17), 3.00 (95%CI: 2.80–3.20), 6.00(95%CI: 5.72–6.29), 3.0 (95%CI: 2.47–3.53) and 3.0(95%CI: 2.80–3.21) months, respectively with significant difference (*p* < 0.001) (Fig. 5a). Additionally, results also showed with the increased number of the other metastatic sites (brain, lung and liver metastasis), the survival significantly decreased, and the median survival for 0, 1 2 and 3 metastatic sites were 5.00 (95% CI: 4.82–5.18), 4.00(95% CI: 3.84–4.16), 3.00 (95% CI: 2.77–3.23) and 3.00 (95% CI: 2.64–3.37) months, respectively (Fig. 5b).

## Discussion

The present study utilized the SEER database and determined the prevalence and associated factors for BM in patients with different histological types of lung cancer. Meanwhile, the quantitative prediction nomogram for different histological types of lung cancer were built.

BM was less frequent in squamous cell carcinoma. However, adenocarcinoma and SCLC exhibited an association with BM, and adenocarcinoma accounted for more than 50% of all the lung cancer patients with BM, a finding that is partly consistent with a hospital-based study [12]. Based on the large population data set provided by SEER, the present study further concluded that small cell lung cancer and lung adenocarcinoma exhibit higher risks of BM than do the other histological subtypes. Previous studies reported similar results. After evaluating 413 patients who were diagnosed with lung cancer, Oliveira et al. found that adenocarcinoma was associated with a higher risk for developing BM, whereas squamous cell cancer was associated with a lower risk of BM [9]. Another study also reported that the most common histological type accompanied by BM was adenocarcinoma [4]. A high prevalence of BM in lung cancer and different BM prevalence rates across various histological subtypes may partly reflect the homogeneity and heterogeneity of lung cancer. Additionally, a cancer registry-based study conducted in Sweden reported that the prevalence of BM in adenocarcinoma was approximately 39%, which was higher than our results. Moreover, although BM was most prevalent among adenocarcinoma, SCLC exhibited a significantly lower prevalence of BM (25%) than did the other lung cancer subtypes, a finding that was different from ours [13]. Accordingly, the homogeneity and heterogeneity of lung cancer may differ according to ethnicity.

In the present study, we also found that different lung cancer histological subtypes exhibited homogeneity and heterogeneity with respect to the factors associated with BM. Regarding homogeneity, we identified three metastatic associated factors for all types of lung cancer: male gender, extrapulmonary metastatic site and lymphatic metastasis.

Among the present cohort, male gender was an associated factor for BM, a finding that was independent of the clinical features of lung cancer. To our knowledge, this is the first report describing sex as a risk factor for BM in lung cancer patients. Lung cancer, unlike breast cancer, is a non-hormone-dependent tumor. The reason that sex may significantly influence metastasis in non-hormone-dependent tumors remains unknown. Further research to determine potential explanations is needed.

The number of metastatic sites was also reported to be one of the metastatic associated factors for all types of lung cancer. According to our analysis, an increase in the number of metastatic sites was associated with an increased prevalence of BM in lung cancer patients. At the same time, for all histological subtypes, a higher grade of lymphatic involvement was associated with an increasing prevalence of BM in lung cancer patients. The aforementioned homogeneous associated factors may help in the surveillance of BM in lung cancer patients. Thus, physicians should focus on their lung cancer patients with these associated factors.

The advantages of early metastasis detection are as follows: 1) less toxic therapy [14, 15]; 2) SREs can be prevented through timely bone-targeted therapy [16]; and 3) improved performance status after therapy [17]. Thus, prompt metastatic screening is necessary. To make an early diagnosis and improve the survival rate of cancer patients, efforts have been made to identify the optimal screening time and method. In the latest studies, bone biomarkers and breast osteoblast-like cells were identified as potential strategies for clinical metastatic screening and early diagnosis [18, 19]. However, the aforementioned strategies require extra techniques and equipment support, which may decelerate the process of clinical screening application. Imaging remains a reliable and accepted strategy for metastatic screening and early diagnosis [20]. To prevent unnecessary radiation exposure and cost, in the present study, a histological type-based prediction nomogram was constructed. A quantitative metastatic risk could be generated utilizing the patient’s sex, lymph node metastasis, extrapulmonary metastatic site number, histological differentiation, and tumor size. We concluded that physicians could perform metastatic screening for their lung cancer patients with a high BM risk.

Inevitably, the present study has several limitations. First, in the present study, only the presence/absence of BM based on the initial diagnosis was analyzed. Disease recurrence or subsequent sites of disease were not provided in the SEER database. Thus, the actual rate of BM in patients with lung cancer might be underestimated. Second, the information on performance status, smoking status, blood type, and body mass index were not provided in the SEER database, which may affect the precision of the predictive nomogram. Third, the present predictive nomogram lacked external validation, more studies are needed in future. Fourth, the site of BM was not recorded in the SEER database, thus it cannot be further identified or analyzed in the study.

## Conclusions

Despite the limitations, the present study provided insight into the epidemiology of BM in patients with newly diagnosed lung cancer, as recorded by the SEER database. The prevalence of BM in lung cancer was 20.9% and different lung cancer histological types showed homogeneous associated factors (male gender, more metastatic sites and more lymphatic metastasis) and heterogeneous associated factors (tumor size and histology differentiation) for BM. The median survival of the bone metastatic lung cancer patients was relatively low. The histological types significantly affect the median survival time of the lung cancer patients with BM. The nomogram has good performance for predicting the BM development in different histological types of lung cancer and the imaging of the skeletal system should be considered to lung cancer patients with a high BM risk.

## Additional files

Additional file 1: **Table S1.** Difference in the prevalence of bone metastasis in different histological subtypes of lung cancer. (PDF 252 kb)

Additional file 2: **Table S2.** Univariate logistic regression for the presence of bone metastases at diagnosis of different subtypes of lung cancer. (PDF 298 kb)

Additional file 3: **Table S3.** Multivariable logistic regression for the presence of bone metastases at diagnosis of lung cancer. (PDF 228 kb)

## Abbreviations

AUC: aura under the curve
BM: Bone metastasis
LCLC: Large cell lung cancer
NCCN: National Comprehensive Cancer Network
NSCLC/NOS: Non-small cell lung cancer/not otherwise specified lung cancer
OR: Odds ratio
ROC: Receiver operating characteristics curve
SCLC: Small cell lung cancer
SEER: Surveillance, Epidemiology, and End Results

## References

[1] Malvezzi M, Bertuccio P, Rosso T (2015). European cancer mortality predictions for the year 2015: does lung cancer have the highest death rate in EU women?. Ann Oncol.

[2] Australian Institute of Health and Welfare (2018). Cancer in Australia: actual incidence data from 1982 to 2013 and mortality data from 1982 to 2014 with projections to 2017. Asia Pac J Clin Oncol.

[3] Al Husaini H, Wheatley-Price P, Clemons M, Shepherd FA (2009). Prevention and management of bone metastases in lung cancer: a review. J Thorac Oncol.

[4] Zhou Y, Yu QF, Peng AF, Tong WL, Liu JM, Liu ZL (2017). The risk factors of bone metastases in patients with lung cancer. Sci Rep.

[5] Kuchuk M, Kuchuk I, Sabri E, Hutton B, Clemons M, Wheatley-Price P (2015). The incidence and clinical impact of bone metastases in non-small cell lung cancer. Lung Cancer.

[6] Bhatia R, Ravulapati S, Befeler A, Dombrowski J, Gadani S, Poddar N (2017). Hepatocellular carcinoma with bone metastases: incidence, prognostic significance, and management-single-center experience. J Gastrointest Cancer.

[7] Makino H, Nishio S, Tsubamoto H (2016). Treatment and prognosis of bone metastasis from cervical cancer (KCOG-G1202s). J Obstet Gynaecol Res.

[8] Network NCC. NCCN Clinical practice guidelines in oncology. Lung Cancer. 2016; https: //www.nccn.org/professionals/physician_gls/pdf /lung.pdf.

[9] Oliveira MB, Mello FC, Paschoal ME (2016). The relationship between lung cancer histology and the clinicopathological characteristics of bone metastases. Lung Cancer.

[10] Conen K, Hagmann R, Hess V, Zippelius A, Rothschild SI (2016). Incidence and predictors of bone metastases (BM) and skeletal-related events (SREs) in small cell lung Cancer (SCLC): a Swiss patient cohort. J Cancer.

[11] Zhang L, Gong Z (2017). Clinical characteristics and prognostic factors in bone metastases from lung Cancer. Med Sci Monit.

[12] Sugiura H, Yamada K, Sugiura T, Hida T, Mitsudomi T (2008). Predictors of survival in patients with bone metastasis of lung cancer. Clin Orthop Relat Res.

[13] Riihimäki M, Hemminki A, Fallah M (2014). Metastatic sites and survival in lung cancer. Lung Cancer.

[14] Curigliano G, Mayer EL, Burstein HJ, Winer EP, Goldhirsch A (2010). Cardiac toxicity from systemic cancer therapy: a comprehensive review. Prog Cardiovasc Dis.

[15] Barth RJ, Gibson GR, Carney PA, Mott LA, Becher RD, Poplack SP (2005). Detection of breast cancer on screening mammography allows patients to be treated with less-toxic therapy. AJR Am J Roentgenol.

[16] LeVasseur N, Clemons M, Hutton B, Shorr R, Jacobs C (2016). Bone-targeted therapy use in patients with bone metastases from lung cancer: a systematic review of randomized controlled trials. Cancer Treat Rev.

[17] Abrahm JL1, Banffy MB, Harris MB. Spinal cord compression in patients with advanced metastatic cancer: "all I care about is walking and living my life". JAMA. 2008;299:937–946. 10.1001/jama.299.8.937 18314436

[18] Brown J, Rathbone E, Hinsley S (2018). Associations between serum bone biomarkers in early breast Cancer and development of bone metastasis: results from the AZURE (BIG01/04) trial. J Natl Cancer Inst.

[19] Scimeca M, Antonacci C, Toschi N, et al. Breast osteoblast-like cells: a reliable early marker for bone metastases from breast Cancer. Clin Breast Cancer. 2017. 10.1016/j.clbc.2017.11.020 Epub ahead of print. 10.1016/j.clbc.2017.11.020 29306659

[20] Guo X, Zhang C, Guo Q (2018). The homogeneous and heterogeneous risk factors for the morbidity and prognosis of bone metastasis in patients with prostate cancer. Cancer Manag Res.

