# Supplementary material for: Nomogram based on homogeneous and heterogeneous associated factors for predicting bone metastases in patients with different histological types of lung cancer
Source: BMC Cancer. 2019 Mar 15;19:238. doi: 10.1186/s12885-019-5445-3 (PMC6420732; doi:10.1186/s12885-019-5445-3)
Supplement: Supplementary file 1 — Table S1. Difference in the prevalence of bone metastasis in different histological subtypes of lung cancer. (PDF 252 kb) [file 12885_2019_5445_MOESM1_ESM.pdf]

Table S1: Difference in the prevalence of bone metastasis in different histological subtypes of lung cancer.

	Adenocarcinoma	Squamous	SCLC	LC	NOS/NSCLC
**Adenocarcinoma**	1.00	χ^2=1552.93 *P*<0.0001	χ^2=0.72 *P*=0.396	χ^2=17.75 *P*<0.0001	χ^2=217.86 *P*<0.0001
**Squamous**	χ^2=1552.93 *P*<0.0001	1.00	χ^2=1120.77 *P*<0.0001	χ^2=89.79 *P*<0.0001	χ^2=429.54 *P*<0.0001
**SCLC**	χ^2=0.72 *P*=0.396	χ^2=1120.77 *P*<0.0001	1.00	χ^2=29.22 *P*<0.0001	χ^2=163.85 *P*<0.0001
**LC**	χ^2=17.75 *P*<0.0001	χ^2=89.79 *P*<0.0001	χ^2=29.22 *P*<0.0001	1.00	χ^2=0.027 *P*=0.87
**NOS/NSCLC**	χ^2=217.86 *P*<0.0001	χ^2=429.54 *P*<0.0001	χ^2=163.85 *P*<0.0001	χ^2=0.027 *P*=0.87	1.00

SCLC=small cell lung cancer; LC=large cell; NOS =not otherwise specified; NSCLC=non-small cell lung cancer.
