# Supplementary material for: Nomogram based on homogeneous and heterogeneous associated factors for predicting bone metastases in patients with different histological types of lung cancer
Source: BMC Cancer. 2019 Mar 15;19:238. doi: 10.1186/s12885-019-5445-3 (PMC6420732; doi:10.1186/s12885-019-5445-3)
Supplement: Supplementary file 2 — Table S2. Univariate logistic regression for the presence of bone metastases at diagnosis of different subtypes of lung cancer. (PDF 298 kb) [file 12885_2019_5445_MOESM2_ESM.pdf]

Table S2: Univariate logistic regression for the presence of bone metastases at diagnosis of different subtypes of lung cancer.

Variable	Patients, No.		Entire cohort									
	No Bone metastasis No (%)	Bone metastasis No (%)	Adenocarcinoma		Squamous		SCLC		LC		NOS/NSCLC	
			OR (95% CI)	*P*-value	OR (95% CI)	*P*-value	OR (95% CI)	*P*-value	OR (95% CI)	*P*-value	OR (95% CI)	*P*-value
**Age (years)**												
18-45	2407(75.7)	774(24.3)	ref	1.0	ref	1.0	ref	1.0	ref	1.0	ref	1.0
46-65	43585(77.2)	12903(22.8)	0.77(0.69-0.86)	<0.001	0.63(0.47-0.84)	0.001	1.31(0.98-1.76)	0.07	0.89(0.49-1.61)	0.69	1.69(1.39-2.05)	<0.001
66-79	51254(81.1)	11968(18.9)	0.61(0.55-0.68)	<0.001	0.50(0.38-0.66)	<0.001	1.20(0.90-1.61)	0.22	0.74(0.41-1.35)	0.33	1.39(1.15-1.70)	0.001
**Sex**												
Female	46496(81.2)	10752(18.8)	ref	1.0	ref	1.0	ref	1.0	ref	1.0	ref	1.0
Male	50750(77.3)	14893(22.7)	1.30(1.25-1.35)	<0.001	1.33(1.23-1.44)	<0.001	1.41(1.31-1.50)	<0.001	1.51(1.21-1.87)	<0.001	1.57(1.46-1.68)	<0.001
**Race**												
White	77539(79.3)	20286(20.7)	ref	1.0	ref	1.0	ref	1.0	ref	1.0	ref	1.0
Black	12781(80.1)	3171(19.9)	0.92(0.87-0.98)	0.005	1.20(1.08-1.33)	0.001	0.73(0.64-0.82)	<0.001	1.00(0.74-1.35)	0.10	1.01(0.92-1.12)	0.83
Asian or Pacific Islander	6136(75.4)	2001(24.6)	1.23(1.15-1.31)	<0.001	1.25(1.06-1.48)	0.008	0.73(0.60-0.90)	0.003	1.70(1.04-2.80)	0.036	1.17(1.02-1.34)	0.02
Indian/Alaska Native	523(80.6)	126(19.4)	1.31(0.99-1.74)	0.057	0.67(0.39-1.15)	0.15	0.61(0.38-0.98)	0.04	1.81(0.47-7.03)	0.39	0.80(0.48-1.35)	0.40
**Marital status**												
Unmarried	15883(78.4)	4386(21.6)	ref	1.0	ref	1.0	ref	1.0	ref	1.0	ref	1.0

Married	76869(79.2)	20146(20.8)	0.97(0.92-1.03)	0.32	0.81(0.74-0.89)	<0.001	1.07(0.97-1.17)	0.19	0.93(0.71-1.21)	0.57	0.91(0.84-0.99)	0.04
**Household income**												
< 50000$	33657(80.1)	8372(19.9)	ref	1.0	ref	1.0	ref	1.0	ref	1.0	ref	1.0
50000-80000$	56393(78.7)	15256(21.3)	1.09(1.04-1.13)	<0.001	1.08(1.00-1.16)	0.05	1.04(0.97-1.11)	0.32	1.04(0.84-1.29)	0.71	0.98(0.91-1.05)	0.52
> 80000$	7190(78.1)	2017(21.9)	1.15(1.07-1.24)	<0.001	1.12(0.96-1.31)	0.16	0.98(0.84-1.14)	0.79	0.65(0.38-1.09)	0.10	0.92(0.80-1.06)	0.25
**Insurance status**												
Uninsured	3751(75.2)	1234(24.8)	ref	1.0	ref	1.0	ref	1.0	ref	1.0	ref	1.0
Insured	92084(79.3)	24031(20.7)	0.80(0.73-0.88)	<0.001	0.65(0.54-0.77)	<0.001	1.09(0.92-1.28)	0.34	0.65(0.42-1.01)	0.051	0.76(0.65-0.89)	0.001
**Metastatic sites**												
0 site	71363(87.5)	10192(12.5)	ref	1.0	ref	1.0	ref	1.0	ref	1.0	ref	1.0
1 site	21085(67.7)	10071(32.3)	3.02(2.89-3.16)	<0.001	4.05(3.73-4.40)	<0.001	2.76(2.56-2.99)	<0.001	3.53(2.78-4.50)	<0.001	3.52(3.26-3.81)	<0.001
2 sites	4339(50.0)	4331(50.0)	6.50(6.07-6.95)	<0.001	9.39(8.15-10.82)	<0.001	5.07(4.56-5.64)	<0.001	7.44(5.34-10.37)	<0.001	7.00(6.25-7.85)	<0.001
3 sites	439(33.6)	868(66.4)	14.87(12.47-17.71)	<0.001	17.71(12.49-25.13)	<0.001	8.34(6.47-10.76)	<0.001	11.50(4.55-29.03)	<0.001	12.57(9.66-16.34)	<0.001
**Tumor size**												
<2 cm	14785(88.1)	1996(11.9)	ref	1.0	ref	1.0	ref	1.0	ref	1.0	ref	1.0
2-5 cm	41463(80.4)	10122(19.6)	1.92(1.79-2.06)	<0.001	1.73(1.45-2.05)	<0.001	1.52(1.32-1.76)	<0.001	1.54(1.02-2.33)	0.04	2.14(1.89-2.41)	<0.001
5-10 cm	23894(77.7)	6859(22.3)	2.48(2.31-2.67)	<0.001	2.40(2.02-2.85)	<0.001	1.62(1.40-1.86)	<0.001	2.28(1.50-3.46)	<0.001	2.67(2.35-3.03)	<0.001

>10 cm	3210(78.6)	875(21.4)	1.93(1.66-2.24)	<0.001	2.67(2.12-3.38)	<0.001	1.63(1.34-1.97)	<0.001	2.09(1.18-3.69)	0.01	2.67(2.17-3.28)	<0.001
**Histological type**													
Well differentiated	6441(94.5)	372(5.5)	ref	1.0	ref	1.0	ref	1.0	ref	1.0	ref	1.0
Moderate differentiated	18285(89.5)	2149(10.5)	2.12(1.86-2.43)	<0.001	0.87(0.63-1.22)	0.43	0.90(0.28-2.89)	0.86	-	1.0	2.94(2.09-4.13)	<0.001
Poor differentiated	26632(82.4)	5681(17.6)	3.31(2.92-3.77)	<0.001	1.58(1.13-2.19)	0.007	1.02(0.41-2.55)	0.96	-	1.0	11.72(8.75-15.70)	<0.001
Undifferentiated	3315(78.5)	909(21.5)	3.98(2.95-5.37)	<0.001	1.89(1.09-3.28)	0.024	1.03(0.42-2.57)	0.94	-	1.0	11.62(8.20-16.47)	<0.001
**Lymphatic metastasis**													
N0	38449(89.9)	4325(10.1)	ref	1.0	ref	1.0	ref	1.0	ref	1.0	ref	1.0
N1	8708(81.4)	1985(18.6)	1.96(1.80-2.12)	<0.001	1.96(1.70-2.26)	<0.001	1.49(1.25-1.78)	<0.001	2.85(1.84-4.39)	<0.001	2.75(2.41-3.15)	<0.001
N2	35358(74.2)	12297(25.8)	3.25(3.09-3.42)	<0.001	3.02(2.75-3.33)	<0.001	1.95(1.73-2.20)	<0.001	3.94(2.90-5.34)	<0.001	3.56(3.26-3.90)	<0.001
N3	12635(69.0)	5682(31.0)	3.95(3.71-4.20)	<0.001	3.87(3.44-4.36)	<0.001	2.73(2.40-3.11)	<0.001	4.96(3.49-7.06)	<0.001	4.28(3.83-4.77)	<0.001

SCLC=small cell lung cancer; LC=large cell; NOS =not otherwise specified; NSCLC=non-small cell lung cancer.
