# Supplementary material for: Nomogram based on homogeneous and heterogeneous associated factors for predicting bone metastases in patients with different histological types of lung cancer
Source: BMC Cancer. 2019 Mar 15;19:238. doi: 10.1186/s12885-019-5445-3 (PMC6420732; doi:10.1186/s12885-019-5445-3)
Supplement: Supplementary file 3 — Table S3. Multivariable logistic regression for the presence of bone metastases at diagnosis of lung cancer. (PDF 228 kb) [file 12885_2019_5445_MOESM3_ESM.pdf]

Table S3: Multivariable logistic regression for the presence of bone metastases at diagnosis of lung cancer.

Variable	No Bone metastasis No (%)	Bone metastasis No (%)	OR (95% CI)	*P*-value
**Age (years)**				
18-45	2407(75.7)	774(24.3)	ref	1.0
46-65	43585(77.2)	12903(22.8)	0.92(0.85-1.00)	0.05
66-79	51254(81.1)	11968(18.9)	0.73(0.67-0.79)	0.73
**Sex**				
Female	46496(81.2)	10752(18.8)	ref	1.0
Male	50750(77.3)	14893(22.7)	1.27(1.23-1.31)	<0.001
**Race**				
White	77539(79.3)	20286(20.7)	ref	1.0
Black	12781(80.1)	3171(19.9)	0.95(0.91-0.99)	0.013
Asian or Pacific Islander	6136(75.4)	2001(24.6)	1.25(1.18-1.31)	<0.001
Indian/Alaska Native	523(80.6)	126(19.4)	0.92(0.76-1.12)	0.41
**Marital status**				
Unmarried	15883(78.4)	4386(21.6)	ref	1.0
Married	76869(79.2)	20146(20.8)	0.95(0.92-0.99)	0.005
**Household income**				
< 50000$	33657(80.1)	8372(19.9)	ref	1.0
50000-80000$	56393(78.7)	15256(21.3)	1.09(1.06-1.12)	<0.001
> 80000$	7190(78.1)	2017(21.9)	1.13(1.07-1.19)	<0.001
**Insurance status**				
Uninsured	3751(75.2)	1234(24.8)	ref	1.0

Insured	92084(79.3)	24031(20.7)	0.79(0.74-0.85)	<0.001
**Metastatic sites**				
0 site	71363(87.5)	10192(12.5)	ref	1.0
1 site	21085(67.7)	10071(32.3)	3.34(3.24-3.45)	<0.001
2 sites	4339(50.0)	4331(50.0)	6.99(6.67-7.33)	<0.001
3 sites	439(33.6)	868(66.4)	13.84(12.32-15.56)	<0.001
**Tumor size**				
<2 cm	14785(88.1)	1996(11.9)	ref	1.0
2-5 cm	41463(80.4)	10122(19.6)	1.81(1.72-1.90)	<0.001
5-10 cm	23894(77.7)	6859(22.3)	2.13(2.02-2.24)	<0.001
>10 cm	3210(78.6)	875(21.4)	2.02(1.85-2.21)	<0.001
**Histological type**				
Well differentiated	6441(94.5)	372(5.5)	ref	1.0
Moderate differentiated	18285(89.5)	2149(10.5)	2.04(1.82-2.28)	<0.001
Poor differentiated	26632(82.4)	5681(17.6)	3.69(3.31-4.12)	<0.001
Undifferentiated	3315(78.5)	909(21.5)	4.75(4.18-5.39)	<0.001
**Lymphatic metastasis**				
N0	38449(89.9)	4325(10.1)	ref	1.0
N1	8708(81.4)	1985(18.6)	2.03(1.91-2.15)	<0.001
N2	35358(74.2)	12297(25.8)	3.09(2.98-3.21)	<0.001
N3	12635(69.0)	5682(31.0)	4.00(3.82-4.18)	<0.001

Abbreviations: SCLC=small cell lung cancer; LC=large cell; NOS =not otherwise specified; NSCLC=non-small cell lung cancer.
